# More light on cancer and COVID-19 reciprocal interaction

**DOI:** 10.1038/s41416-020-01246-0

**Published:** 2021-02-03

**Authors:** Patrick Brest, Baharia Mograbi, Paul Hofman, Gerard Milano

**Affiliations:** 1 Université Côte d’Azur, IRCAN, Centre Antoine Lacassagne, CNRS, Inserm, FHU OncoAge, 06107 Nice, France; 2 Université Côte d’Azur, CHU-Nice, Laboratory of Clinical and Experimental Pathology, FHU OncoAge, Hospital-Integrated Biobank (BB-0033-00025), 06001 Nice, France; 3 Université Côte d’Azur, Centre Antoine Lacassagne, UPR7497, 06100 Nice, France

**Keywords:** Cancer epidemiology, Risk factors

## Abstract

Cancer patients are vulnerable to COVID-19 with consequences on treatment delays and on mortality rate. This Comment explores the interaction between COVID-19 and cancer with attention paid to the modulation by cancer treatments of both ADAM17 and TMPRSS2, the proteases which control ACE2 processing, the SARS-CoV-2 target.

## Main

**Fig. 1:**
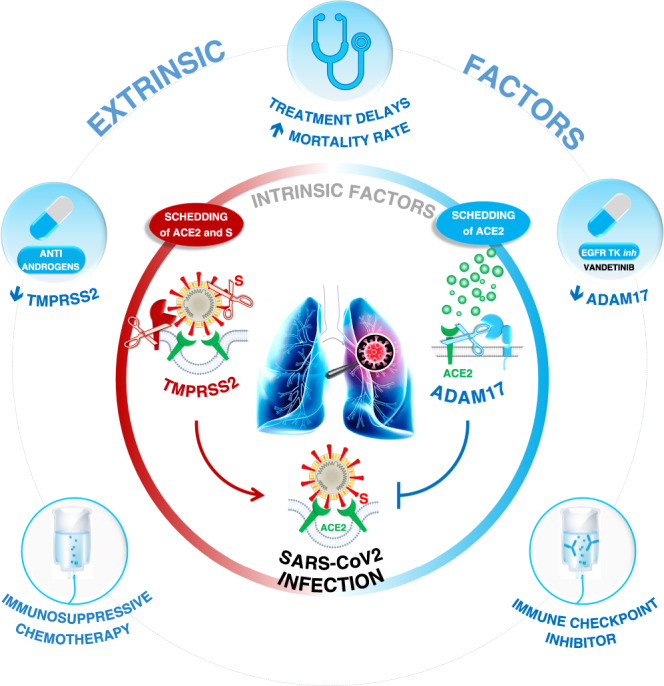
Reciprocal interactions between COVID-19 and cancer. COVID-19 infectiousness can be majored by an increase in TMPRSS2 under the presence of anti-androgen therapy or by a vandetanib-mediated decrease in ADAM17 expression. On the other hand, immunosuppressive chemotherapy and immune checkpoint inhibitors may contribute to worsening to COVID-19-related symptoms.

The severe acute respiratory syndrome coronavirus-2 (SARS-CoV-2) pandemic is placing a huge burden on public health and is impacting civil society and the global economy. There is a burning need for more knowledge regarding the mechanisms governing SARS-CoV-2 infectiousness. The spike (S) glycoprotein mediates SARS-CoV-2 cellular entry via binding to angiotensin-converting enzyme 2 (ACE2) following proteolysis by transmembrane protease serine 2 (TMPRSS2). However, more light is needed regarding the mode of acquisition and possible origins of the variable clinical spectrum of COVID-19. Recent experimental results do not support the current dogma that dual measurement of ACE2 and TMPRSS2 expression may fully describe cell infectivity. In fact, as recently reported by others^1^ and us,^2^ ACE2 processing at membrane level involves at least two proteases with TMPRSS2 regulating ACE2-SARS-CoV-2 cell entry as well as disintegrin and metalloproteinase domain-containing protein 17 (ADAM17), which acts in an opposite way to TMPRSS2 by inducing ACE2 shedding (Fig. 1). In brief, TMPRSS2 cleaves not only ACE2, but also the SARS-CoV-2 S protein, thus participating in the membrane fusion and cellular uptake of the virus. In contrast, ADAM17 acts directly on ACE2 and leads to the release of ACE2-cleaved protein into the extracellular cellular space. Competition between these two proteases for ACE2 processing has previously been reported by Heurich et al.^1^ Promising recent studies point towards a soluble human recombinant isoform or a molecularly modified ACE2 protein as potential therapeutic tools to counter the spread of SARS-CoV-2.^3^ Consequently, when the viral load is high, one can hypothesise that the shedding barrier effect conferred by the ADAM17–ACE2 interaction may be overwhelmed, thus facilitating subsequent infection. Considering these opposite roles of ADAM17 and TMPRSS2 in ACE2 processing and SARS-CoV-2 infectivity, the next step is to determine the factors controlling their respective expressions. We recently reported the role of germinal genetic polymorphisms at this level.^2^ In brief and based on a broad public database compilation, we suggested that germinal polymorphisms may regulate the expression of the SARS-CoV-2 cellular target itself and that of ADAM17 and TMPRSS2 proteases.

Cancer patients are particularly vulnerable to the risk of COVID-19 with consequences on treatment delays and on increased mortality rate.^4^ More effort is needed to raise the bar still further in the areas of research exploring the interactions between COVID-19 and cancer. First, and quite logically, one can advocate the potential negative impact of COVID-19 on malignant disease evolution, including detrimental treatment restrictions.^5^ However, more consideration should also be given to the repercussions of cancer on COVID-19, including the impact of cancer treatments, and particular attention should thus be paid to the modulation of the above-highlighted intrinsic key factors which are both ADAM17 and TMPRSS2 (Fig. 1). TMPRSS2 plays a key role in prostate cancer and its gene expression is strongly upregulated by androgens.^6^ Thus, anti-androgens widely used in prostate cancer treatment can downregulate TMPRSS2 cell expression thus providing relative protection of this category of (generally elderly) patients for SARS-Cov-2 vulnerability, as recently reported by Montepoli and co-workers.^7^ Also, the extent to which TMPRSS2 expression can be modulated by other anti-cancer treatments remains to be explored. On the other hand, ADAM17 cellular activity has been shown to be positively regulated by epidermal growth factor receptor (EGFR) signalling,^8^ thus raising the question of the impact of anti-EGFR tyrosine kinase inhibitors (TKI) on COVID-19 susceptibility through ADAM17 down-tuning (Fig. 1). Indeed, ADAM17 expression has been shown to be downregulated by the application of vandetanib, an EGFR TKI in clinical use.^9^ Apart from the above-mentioned effects of cancer treatments on the two proteases regulating ACE2 processing, other extrinsic variables may also play a significant role in virus infectiousness. This is true for immunosuppressive chemotherapy and cellular therapeutics which can render treated patients more vulnerable to SARS-CoV-2 (Fig. 1). The interaction between immune checkpoint inhibitor (ICI) use and COVID-19 needs greater attention. In particular, ICI-mediated cytokine release may represent a real clinical issue in the early phase of COVID-19 with a worsening of its induced morbidity and complicating the clinical course. An exacerbation of ICI-related lung injury can result from triggered immune deregulation by T cell hyperactivation. This can in turn facilitate acute respiratory distress syndrome which is a dreaded COVID-19 complication (Fig. 1).^10^

Anti-androgen treatment, anti-EGFR TKI and ICI are major therapeutic tools in cancer which, as suggested above, can impact key molecular actors of COVID-19 infectiousness (Fig. 1). More attention is encouraged to identify other molecular interfaces between cancer and COVID-19 in order to shed further light on the complex and highly clinically relevant interaction between these two diseases. This need must be carefully evaluated by considering the potential impact of cancer on SARS CoV-2 vulnerability during both initiation and later treatment phases, including clinical trials.

## Data Availability

Not applicable.
